# Potent dual MAGL/FAAH inhibitor AKU-005 engages endocannabinoids to diminish meningeal nociception implicated in migraine pain

**DOI:** 10.1186/s10194-023-01568-3

**Published:** 2023-04-11

**Authors:** Adriana Della Pietra, Georgii Krivoshein, Konstantin Ivanov, Raisa Giniatullina, Henna-Kaisa Jyrkkänen, Ville Leinonen, Marko Lehtonen, Arn M. J. M. van den Maagdenberg, Juha Savinainen, Rashid Giniatullin

**Affiliations:** 1 A.I. Virtanen Institute for Molecular Sciences, University of Eastern Finland, Kuopio, Finland; 2 Department of Human Genetics, Leiden University Medical Center, Leiden, The Netherlands; 3 Institute of Biomedicine, University of Eastern Finland, Kuopio, Finland; 4 Department of Neurosurgery, Kuopio University Hospital and Neurosurgery, Institute of Clinical Medicine, University of Eastern Finland, Kuopio, Finland; 5 School of Pharmacy, Faculty of Health Sciences, University of Eastern Finland, Kuopio, Finland; 6 Department of Neurology, Leiden University Medical Center, Leiden, The Netherlands

**Keywords:** Headache, Anandamide, Neuronal firing, 2-arachidonoylglycerol

## Abstract

**Background:**

Engaging the endocannabinoid system through inhibition of monoacylglycerol lipase (MAGL) and fatty acid amide hydrolase (FAAH), degrading endocannabinoids (endoCBs) 2-arachidonoylglycerol (2-AG) and anandamide (AEA), was proposed as a promising approach to ameliorate migraine pain. However, the activity of MAGL and FAAH and action of endoCB on spiking activity of meningeal afferents, from which migraine pain originates, has not been explored thus far. Therefore, we here explored the analgesic effects of endoCB enhancement in rat and human meningeal tissues.

**Methods:**

Both MAGL and FAAH activity and local 2-AG and AEA levels were measured by activity-based protein profiling (ABPP) and LC–MS/MS, respectively, in rat meninges obtained from hemiskulls of P38-P40 Wistar rats and human meninges from elderly patients undergoing non-migraine related neurosurgery. The action on endoCBs upon administration of novel dual MAGL/FAAH inhibitor AKU-005 on meningeal afferents excitability was tested by investigating paired KCl-induced spiking and validation with local (co-)application of either AEA or 2-AG. Finally, the specific TRPV1 agonist capsaicin and blocker capsazepine were tested.

**Results:**

The basal level of 2-AG exceeded that of AEA in rat and human meninges. KCl-induced depolarization doubled the level of AEA. AKU-005 slightly increased spontaneous spiking activity whereas the dual MAGL/FAAH inhibitor significantly decreased excitation of nerve fibres induced by KCl. Similar inhibitory effects on meningeal afferents were observed with local applications of 2-AG or AEA. The action of AKU-005 was reversed by CB1 antagonist AM-251, implying CB1 receptor involvement in the anti-nociceptive effect. The inhibitory action of AEA was also reversed by AM-251, but not with the TRPV1 antagonist capsazepine. Data cluster analysis revealed that both AKU-005 and AEA largely increased long-term depression-like meningeal spiking activity upon paired KCl-induced spiking.

**Conclusions:**

In the meninges, high anti-nociceptive 2-AG levels can tonically counteract meningeal signalling, whereas AEA can be engaged on demand by local depolarization. AEA-mediated anti-nociceptive effects through CB1 receptors have therapeutic potential. Together with previously detected MAGL activity in trigeminal ganglia, dual MAGL/FAAH inhibitor AKU-005 appears promising as migraine treatment.

**Graphical Abstract:**

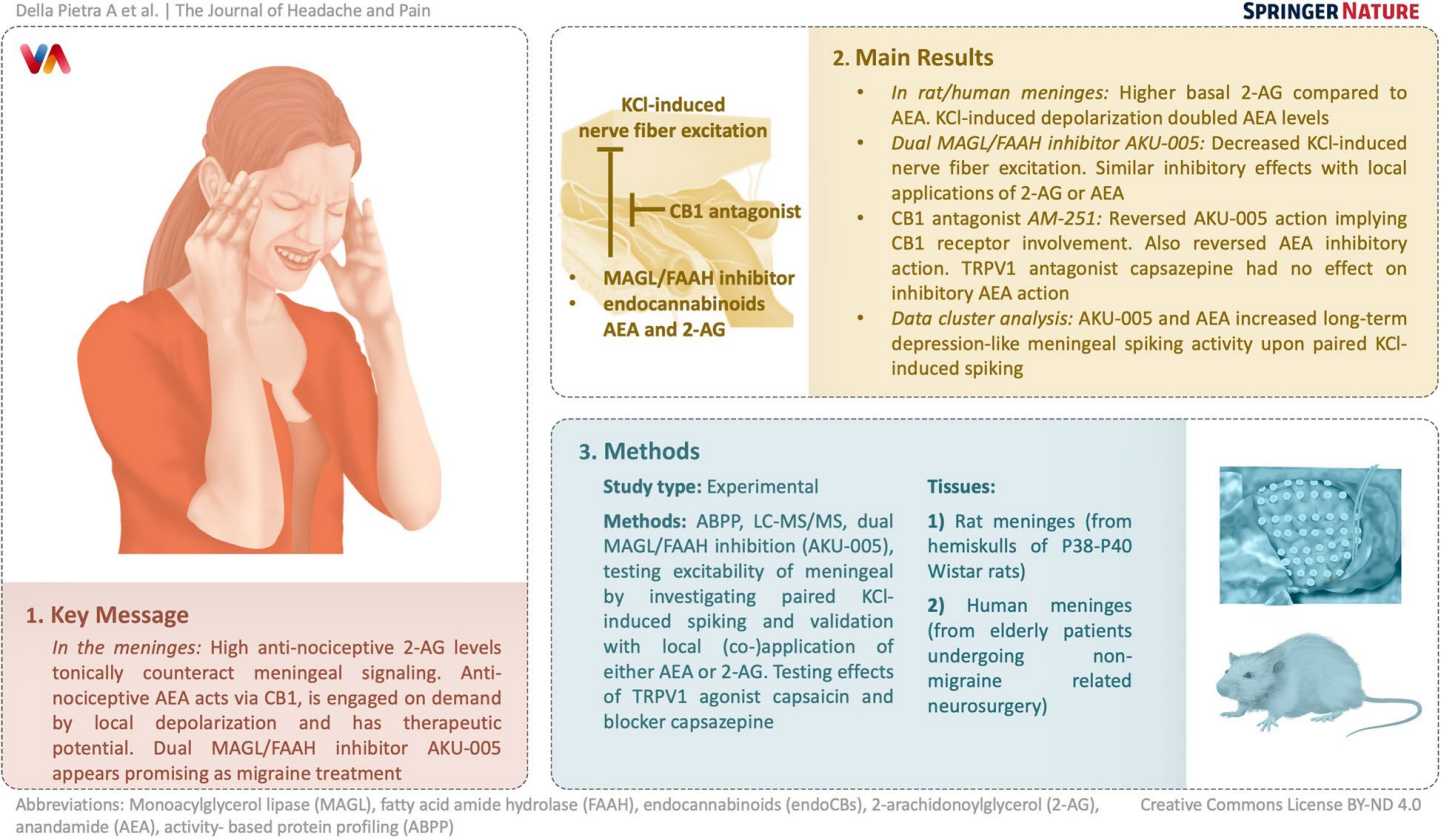

**Supplementary Information:**

The online version contains supplementary material available at 10.1186/s10194-023-01568-3.

## Background

Migraine is a common brain disorder that is characterized by a throbbing pain of generally unilateral nature, as well as other neurological symptoms that may vary between patients [1, 2]. Despite continuous debate about the relative contribution of the central and peripheral nervous system in generating migraine attacks [2, 3], there is compelling evidence that activation of the peripheral part of the trigeminovascular system, which main components are the trigeminal ganglia and the peripheral afferents of the trigeminal nerve in the meninges, is of pivotal importance in triggering headache mechanisms [4]. Recent observations revealed that nociceptive signalling in these pain-related areas is distinctly sensitive to various neuromodulators [5–7], including emerging endogenous anti-nociceptive compounds known as endocannabinoids (endoCBs) [8].

Components of the endocannabinoid system, which are intrinsically active in our body, may influence trigeminovascular system nociceptive processing [9]. Engaging the endocannabinoid system has already been proposed as approach to produce analgesia in various pain types [10–12]. The two main endoCBs, anandamide (*N*-arachidonoylethanolamide, AEA) and 2-arachidonoylglycerol (2-AG) act on cannabinoid type 1 (CB1) receptors, mainly expressed on neurons, and type 2 (CB2) receptors that are mainly expressed on immune cells [13, 14]. While 2-AG acts as cannabinoid agonist activating both CB1 and CB2 receptors [15], AEA, in addition to acting on CB1/2 receptors, activates the transient receptor potential vanilloid 1 (TRPV1) [16], thus adding complexity to the pain mechanisms. One may, therefore, envisage that migraine pain can be treated by enhancing the levels of AEA and 2-AG by inhibiting its degrading serine hydrolases enzymes fatty acid amide hydrolase (FAAH) and monoacylglycerol lipase (MAGL), respectively [17, 18]. The distinct regional distribution of MAGL and FAAH within the trigeminal nociceptive system, strongly supports the importance of dual inhibition of both enzymes. Of relevance is that dual MAGL-FAAH inhibitor AKU-005 was previously shown to be potent in inhibiting endoCBs hydrolases in various areas of the nervous system implicated in migraine pain signalling [19, 20]. In a previous study, we demonstrated that rat trigeminal ganglia have an exceptionally high level of MAGL activity compared to a very low activity of FAAH [20]. The endoCBs profile of meningeal areas, which are innervated primarily by peripheral afferents of trigeminal ganglia neurons [21], has, however, not been studied. Therefore, we here aim to determine the functional role of endoCBs AEA and 2-AG, as well as MAGL and FAAH enzymes activity, in rat meninges high potassium-induced spiking activity. Notably, it was previously observed that only pain sensation is evoked after stimulation of dura mater during head surgery (as reviewed in [22, 23]) suggesting that meningeal afferents contain mainly nociceptive fibres. We used KCl stimulation, which presumably activates these meningeal nociceptive fibres and mimics electrical stimulation used in human studies. Moreover, meningeal application of high potassium, to some extent also simulates what happens during cortical spreading depolarization, which is known to be associated with large bursts of potassium ions [24] and that can lead to prolonged activation of different types of dural afferents [25, 26]. Indeed, for better translation of our findings to humans, we also performed a pilot endocannabinoid system profiling in human meninges.

Our data show exactly the opposite endocannabinoid system profile in meninges compared to the one previously uncovered in trigeminal ganglia [20]. Therefore, there is an unusual reciprocal relationship in endocannabinoid system between two key parts of the peripheral sensory system that are pivotal in migraine pain signalling. Our findings strongly support the benefit of dual MAGL/FAAH inhibition to ensure the most efficient analgesia via engagement of both main endoCBs, 2-AG and AEA, in various parts of the peripheral trigeminovascular system to treat migraine pain.

## Material and methods

### Animals

P24-P26 male and female outbred Wistar rats (Envigo Laboratories B.V., The Netherlands) were delivered to the Lab Animal Centre of University of Eastern Finland. After a 2-week quarantine rats were used at P38-P40 for experimentation. For testing MAGL/FAAH activity and measuring 2-AG and AEA levels, meninges from 6 rats were used, where “*N*” refers to the number of rat meninges. For the electrophysiology recordings from meningeal trigeminal afferents of hemiskulls, 35 rats were used, where “*N*” refers to the number of hemiskulls (2 per rat). Animals were housed under the following conditions: 12-h dark/light cycle, grouped housing, ad libitum access to food and water, ambient temperature of 22°C. All experimental procedures performed in this study follow the rules of the European Community Council Directive of September 22, 2010 (2010/63/EEC). The Animal Care and Use Committee of the University of Eastern Finland (licence EKS-008–2019) approved all experimental protocols.

### Rat meninges

Naïve rat meninges were tested both in pure artificial cerebrospinal fluid (aCSF composed by 120 NaCl, 2.5 KCl, 2 CaCl_2_, 1 MgCl_2_, 11 glucose, 24 NaHPO_4_ and 30 NaHCO_3_, bubbled with 95% O_2_ / 5% CO_2_ at room temperature (RT) with pH maintained at 7.25 – 7.35 (control) and in a combined solution of KCl and aCSF (test condition). Meningeal samples were maintained for 4 h in either aCSF or a solution of 100 mM KCl in oxygenated artificial cerebrospinal fluid (aCSF), containing (in mM): 22.5 NaCl, 100 KCl, 2 CaCl_2_, 1 MgCl_2_, 11 glucose, 24 NaHPO_4_ and 30 NaHCO_3_, bubbled with 95% O_2_ / 5% CO_2_ at the physiological temperature of 37 °C with pH maintained at 7.25 – 7.35. Rat meninges were then stored at -80°C. Each meningeal sample was homogenized and used for both activity-based protein profiling (ABPP) and mass spectrometric (LC–MS/MS) analysis of 2-AG and AEA.

### Human meninges

Human meningeal tissues, in particular dura mater components of ~ 2 cm × 2 cm × 2 mm (exact shape and size was dependent on the surgery), were collected from six female and six male 50- to 70-year-old patients undergoing elective neurosurgery for removal of a meningioma or clipping of an unruptured aneurysm. The meningeal tissue was extracted through the craniotomy frontal to right Sylvian fissure and frozen immediately after extraction with liquid nitrogen in operating room and then stored at -80°C. Written informed consent was obtained from all subjects before the study. The ethics committee approval number license approving the surgical removal of human dura samples is 164/2016, 22.6.2021.

### Activity-based protein profiling of MAGL and FAAH activity

Human and rat meningeal samples were mechanically homogenized in phosphate-buffered saline (PBS) and protein concentrations were determined with the bicinchoninic acid (BCA) protein assay, as described previously [27]. Competitive activity-based protein profiling (ABPP) using tissue homogenates was conducted to visualize the selectivity of inhibitors toward endoCB hydrolases FAAH and MAGL and against other serine hydrolases in tissue proteomes. We used the active site serine‐targeting fluorescent fluorophosphonate probe TAMRA‐FP (ActivX Fluorophosphonate Probes, Thermo Fisher Scientific Inc., Rockford, IL, USA), as described previously [28]. Briefly, tissue homogenates were pre-treated for 1 h with dimethyl sulfoxide (DMSO) or with selected MAGL-inhibitor JJKK-048 (100 nM, Cayman chemicals, Ann Arbor, MI, USA) and KML-29 (1 μM, Cayman chemicals) or with FAAH-inhibitor JZP-327A (1 μM, synthetized at University of Eastern Finland, Kuopio, Finland) and JZP-430 (1 μM synthetized in University of Eastern Finland, Finland) or dual MAGL/FAAH inhibitor AKU-005 (100 nM synthetized in University of Eastern Finland, Finland). Subsequently, TAMRA‐FP was added and incubated for 1 h at RT (final probe concentration 2 μM) to label active serine hydrolases. The reaction was then quenched by adding 2X gel loading buffer, after which 10 μg protein was loaded per lane and the proteins were resolved in 10% SDS‐PAGE together with molecular weight standards. TAMRA‐FP labelled proteins were visualized by ChemiDoc™ MP imaging system (BIO-RAD Laboratories, Hercules, CA, USA) with Cy3 blot application (602/50, Green Epi, Manual Exposure 10 – 120 s). Quantification of bands was performed by the software ImageLab (BIO-RAD Laboratories).

### Quantification of endoCBs by liquid chromatography coupled with triple quadrupole mass spectrometry

Sample preparation and instrumental parameters for analysis of endoCBs levels have been previously described in detail [29]. In brief, prior to the instrumental analysis, samples were extracted with a solution of methanol, chloroform and water. Samples were measured with a reversed phase liquid chromatography technique coupled with the triple quadrupole mass spectrometry (LC–MS/MS), operating in multiple reaction monitoring scanning (MRM). LC–MS/MS analysis was performed using Agilent Technologies 6410 triple quadrupole mass spectrometer coupled to Agilent Technologies 1200 series HPLC system. Separation was achieved using Zorbax Eclipse XDB-C18 (Agilent Technologies) column with an isocratic elution. The flow rate was 0.5 mL/min, and the total run time was 4 min. The mass spectrometer was operated in positive ion mode using MRM for quantification of the analytes. Deuterated internal standards (AEA-d8 and 2-AG-d8) were used for quantification. This method is selective, precise, and accurate for concentrations within a range of 0.4 – 70 nM for N-acylethanolamines and 40 – 11,000 nM for 2-AG.

### Electrophysiology

Rat hemiskull preparations for direct spike recordings from meningeal trigeminal afferents were prepared, as previously described [30, 31]. Briefly, after CO_2_ asphyxiation and decapitation, the skin and muscles were removed from the skull, which was dissected mid-sagittal in two hemiskulls. Next, both hemiskulls were gently separated from the brain hemispheres leaving the innervated meninges inside hemiskulls intact. A cleaning procedure was carried out within 10 – 15 min using oxygenated aCSF. Afterwards, isolated hemiskulls were placed in a recording chamber that was continuously perfused with aCSF (6 – 7 mL/min) and oxygenated with 95% O_2_ / 5% CO_2_ mixture. The distal part of the nervus spinosus located between the trigeminal ganglion and intercross with the middle meningeal artery was cleaned from surrounding meninges and cut 1 mm before its entry into the trigeminal ganglion using a 30G needle. This part of the nerve was inserted into a borosilicate glass microcapillary (GC150F-10, Harvard apparatus, Edenbridge, UK), which was filled with aCSF and connected to the recording electrode. The inserted isolated nerve generated a tight seal by light suction in the grass capillary. Spiking activity from nerve fibers was recorded using a low-noise digital amplifier (ISO 80, World Precision Instruments, Sarasota, FL, USA) with a gain of 10,000X and a bandpass of 300 – 3,000 Hz. Signals were obtained and digitized at 8-µsec intervals using a NIPCI-6221 data acquisition board (National Instruments, Austin, TX, USA). Electrical signals were visualized with the WinEDR V3.4.6 software (Strathclyde University, UK) and analysed with MATLAB-based software [30]. In all experiments, to stabilise the baseline, 10 min of spontaneous action potential activity was recorded at the beginning of the experiment. After that, two subsequent 50 mM KCl applications (‘paired KCl pulses’) with compensated osmolarity were applied for 10 min to induce general nerve excitability. Twenty minutes of washout with aCSF were done between the pulses. At the end of each experiment, capsaicin was applied for 10 min (1 µM, Tocris Bioscience, Bristol, UK) to measure the output of neuronal activity from TRPV1 receptors. Recordings were performed from 7 experimental groups with 5 – 8 hemiskulls each. The first set of experiments (group 1) served as a control where DMSO was applied within the same timeline and concentration as the used compounds that were diluted in DMSO. Note that the same control group data was compared with data of other experimental groups (and, therefore, reappears in grey in the figures). For the next three experimental groups, single compounds were tested, *i.e.* dual MAGL/FAAH inhibitor AKU-005 (100 nM) (group 2), AEA (10 µM) (group 3), or 2-AG (10 µM) (group 4) were applied within 10 min of the 2^nd^ of the paired KCl pulses with an additional 10-min recording pre and post application of KCl. For the last three experimental groups, combinations of compounds were tested, *i.e.* AKU-005 + the specific CB1 antagonist and inverse agonist AM-251 (1 µM, Tocris Bioscience/Biotechne Abingdon, UK) (group 5), AEA + AM-251 (group 6), or AEA + the specific TRPV1 blocker capsazepine (20 µM, Tocris Bioscience) (group 7), were applied during the 2^nd^ of the paired KCl pulses. Of note, AM-251 was pre-applied 10 min before AKU-005 or AEA, while capsazepine was applied immediately after 10 min of recording of spontaneous action potential activity. All drugs were diluted to their final concentration in aCSF immediately before usage and were applied closely to the receptive field (*i.e.* the intersection of the meningeal artery and trigeminal nerve branch) by fast perfusion (6 – 7 mL/min). Electrophysiological results are presented as either the sum of action potentials (APs) for the specific timeline or as the ratio between the sum of APs for the specific timeline before and after drug applications within an experimental group. Finally, a comparison was performed based on either the sum of APs or the ratio of APs between different experimental conditions, *i.e.* control, single compounds, or combination of compounds.

### Cluster analysis

After filtering the original recordings at 100 – 9,000 Hz using a IIR Chebyshev-type two-filter for AP detection, an advanced cluster analysis was performed, similar to what was previously described [30, 32, 33]. The background noise of the setup was first assessed for 20 s in the absence of APs to determine the correct threshold for the detection of APs. We selected as APs two-phase signals within the range of 0.3 – 1.8 ms whose amplitude exceeded 5 standard deviations (SD) of the baseline noise level. APs amplitudes were normalized to baseline noise and expressed as SD values arbitrary units (a.u.). For each AP, the rise and decay time, the positive and negative amplitude phases, AP areas, and their total duration were calculated using MATLAB (MathWorks). To automatically recognize the most compact groups of APs (clusters), the ‘Klusta-Kwik’ application [34] was used. Positive phase of AP amplitude *vs.* AP duration were used as input parameters for clusterization. Using this approach, the total flow of APs was separated into 8 to 34 individual clusters for each experiment, where “n” refers to the number of clusters. Next, all clusters within one experiment were divided into paired-pulse potentiation (PPP) and paired-pulse depression (PPD) groups based on the ratio of APs evoked by the 1^st^ and 2^nd^ KCl pulse. When the number of APs during the 1-min active phase of the 2^nd^ KCl pulse exceeded the number of APs induced within the 1-min active phase of the 1^st^ KCl pulse, the cluster was considered PPP-related. In case the ratio of APs within described timing was the opposite a cluster was placed in the PPD group. This cluster analysis was repeated for control experiments and for experiments testing the effects of single compounds AKU-005, AEA, and 2-AG.

### Experimental design and analysis

Experiments had a randomized design. The electrophysiological recordings were not blinded as the same researcher performing the experiment also carried out the analysis. However, the analysis was automatically carried out by using MATLAB (MathWorks), preventing any personal data interpretation. ABPP and mass spectrometry experiments were blinded. Group size was calculated according to our previous experience with the same experimental techniques. The data were analysed and plotted using Origin Pro (Origin Lab Corporation, Northampton, MA, USA) and Graph Pad Prism (GraphPad Prism Software, San Diego, CA, USA). The resulting data are presented as the mean ± standard error of the mean (m ± SEM). Distribution normality was assessed by the Anderson–Darling test. After that, based on whether data were paired or not, the Mann–Whitney test or Wilcoxon non-parametric tests for single comparisons and Kruskal–Wallis or Friedman non-parametric tests with Dunn's multiple comparisons were used. The significance level was set at *p* < 0.05.

## Results

### Profiling MAGL and FAAH activity and endoCB levels in rat meninges

**Fig. 1:**
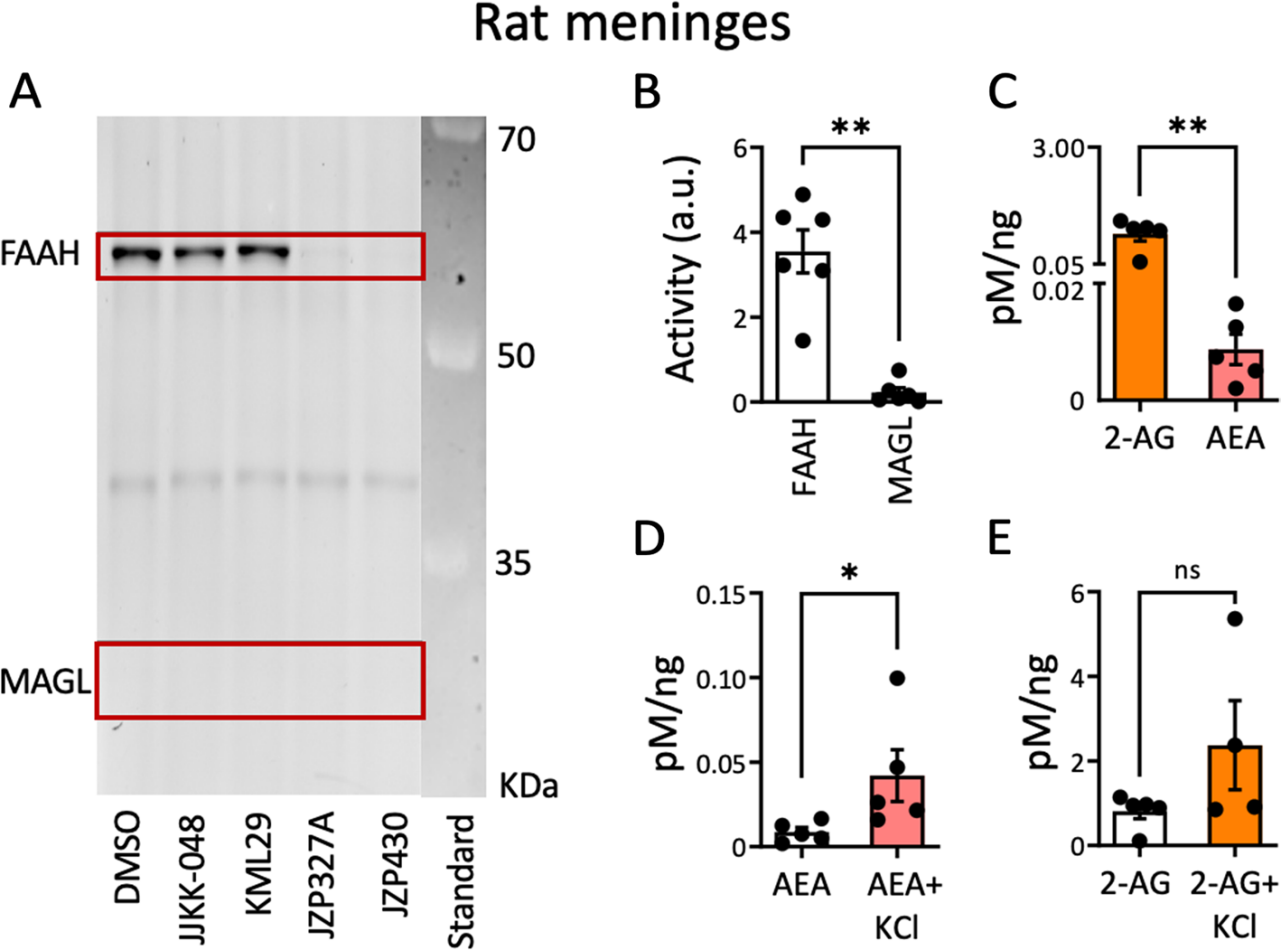
Competitive gel-based ABPP reveals higher FAAH over MAGL activity in rat meninges and LC–MS/MS shows 2-AG as the main meningeal endoCB. **A** Rat meninges were pre-incubated for 1 h with DMSO (vehicle), MAGL-inhibitors JJKK-048 (100 nM) and KML-29 (1 μM) and FAAH-inhibitors JZP-327A (1 μM) and JZP-430 (1 μM), and then labelled with fluorescent probe TAMRA-FP, as indicated in the Methods. TAMRA-FP-labelled bands appear dark after in-gel imaging. FAAH and MAGL were identified based on selective inhibition and their expected molecular weights. MAGL and FAAH band-intensities after DMSO treatment represent basal MAGL and FAAH activities, respectively. Note that FAAH activity after DMSO treatment is high, whereas basal MAGL activity is practically absent. Basal FAAH- activity was reduced with JZP-327A (*N* = 6, Friedman test) and with JZP-430 (*N* = 6, Friedman test). MAGL-inhibitors JJKK-048 (100 nM) and KML-29 (1 μM) did not affect FAAH basal activity. **B** Statistics comparing the basal activity of MAGL and FAAH in rat meninges. Basal FAAH activity was higher than MAGL activity (*N* = 6, Mann Whitney U test, ** = 0.002). **C** LC–MS/MS data comparing AEA and 2-AG levels in naïve rat meninges. The level of 2-AG was much higher than that of AEA (*N* = 5, Mann Whitney U test, ** = 0.002). **D** LC–MS/MS data comparing AEA levels in naïve rat meninges vs. after 4 h incubation of 100 mM KCl pro-nociceptive treatment. KCl treatment significantly increased AEA levels compared to its basal level (*N* = 5, Mann Whitney U test, * = 0.033). **E** LC–MS/MS data comparing 2-AG levels in naïve rat meninges (*N*=5) vs. after a 4-h 100-mM KCl pro-nociceptive treatment (*N*=4). No significant differences in 2-AG levels were detected (Mann Whitney U test)

To profile endocannabinoid system factors in male rat meninges, we first assessed with ABPP the in vitro activity and inhibition of main endoCBs hydrolyzing enzymes MAGL and FAAH (Fig. 1A). Basal activity of FAAH (DMSO treatment; Fig. 1A and B) was considerably higher (3.5 ± 0.5 a.u., *N* = 6) than MAGL activity (0.2 ± 0.1 a.u., *N* = 6, *p* = 0.002, Mann Whitney U test; Fig. 1B). FAAH activity was inhibited by FAAH-inhibitors JZP-327A and JZP-430 at micromolar concentrations (Fig. 1A). In contrast, MAGL-inhibitors KML-29 (1 μM) and JJKK-048 (100 nM) did not affect FAAH basal activity in rat meninges (Fig. 1A). The novel dual MAGL/FAAH inhibitor AKU-005, at the low concentration of 100 nM, significantly reduced FAAH basal activity in rat female meninges to 72.5 ± 3.1% (*N* = 5, *p* = 0.002, Mann Whitney U test; Additional file 1) in comparison to FAAH basal activity in DMSO (taken as 100%). Using quantitative LC–MS/MS analysis, we also measured 2-AG and AEA levels in rat meninges. In line with the profiled enzymatic activities, the level of 2-AG (0.8. ± 0.2 pM/ng, *N* = 5) was considerably higher than that of AEA (0.009 ± 0.003 pM/ng, *N* = 5, *p* = 0.008, Mann Whitney U test; Fig. 1C). When neurons were depolarized by application of KCl, the level of AEA increased from 0.009 ± 0.003 pM/ng (*N* = 5) to 0.04 ± 0.02 pM/ng (*N* = 5, *p* = 0.016, Mann Whitney U test; Fig. 1D), whereas the level of 2-AG, which was already naturally high, had an ambiguous apparent increase in some animals, but this did not reach statistical significance (Fig. 1E). The results indicate that FAAH activity in rat meninges is high and that the AEA level is sensitive to KCl-treatment, whereas MAGL activity is virtually absent in the meningeal preparation and the level of 2-AG does not seem to alter by application of KCl.

### Profiling MAGL and FAAH activity and endoCB levels in human meninges

**Fig. 2:**
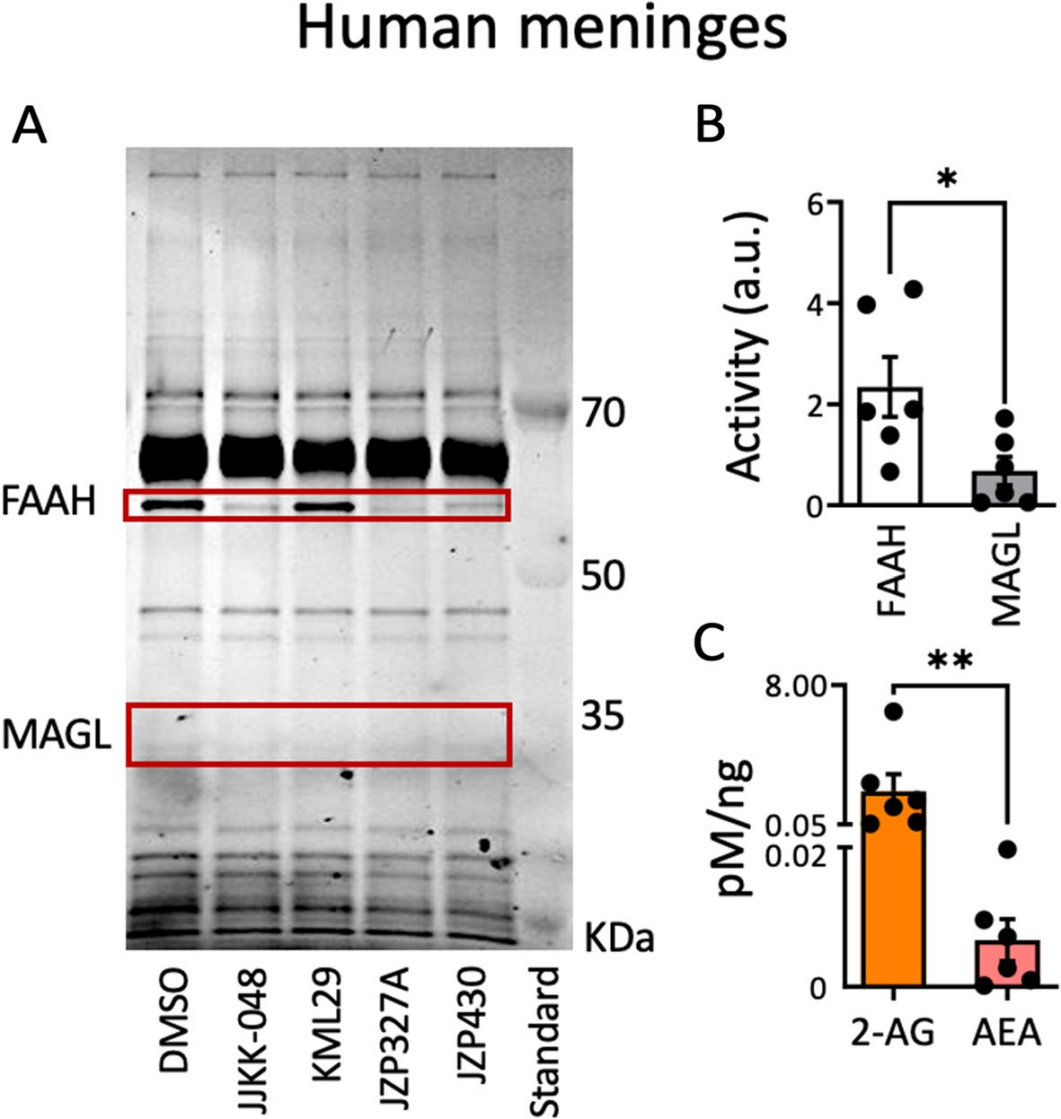
Competitive gel-based ABPP reveals high FAAH over MAGL activity in human dura mater. **A** Human dura mater was pre-incubated for 1 h with DMSO (vehicle), MAGL-inhibitors JJKK-048 (100 nM) and KML-29 (1 μM) and FAAH-inhibitors JZP-327A (1 μM) and JZP-430 (1 μM), and then labelled with fluorescent probe TAMRA-FP, as indicated in the Methods. TAMRA-FP-labelled bands appear dark after in-gel imaging. FAAH and MAGL were identified based on selective inhibition and their expected molecular weights. MAGL and FAAH band-intensities after DMSO treatment represent basal MAGL and FAAH activities, respectively. Note that FAAH activity after DMSO treatment is high whereas basal MAGL activity is practically absent. Basal FAAH- activity appears reduced with JZP-327A and with JZP-430. MAGL-inhibitor, JJKK-048 (100 nM), decreased FAAH basal activity as off-target. **B** Statistics comparing the basal activity of MAGL and FAAH in human meninges. FAAH basal activity (DMSO) was found higher than MAGL activity (*N* = 6, Mann Whitney U test, * = 0.033). **C** LC–MS/MS data comparing AEA and 2-AG levels in naïve human meninges. The level of 2-AG was much higher than that of AEA (*N* = 6, Mann Whitney U test, ** = 0.002)

To evaluate the translational potential of our findings to the human situation, we also profiled corresponding endocannabinoid system players in human meninges. Human meninges, in particular from dura mater, were collected from patients undergoing neurosurgery for unrelated pathologies, such as the removal of a meningioma or clipping of an aneurysm. As was done for rat meninges, first MAGL and FAAH activity were assessed using ABPP (Fig. 2A). Again, basal FAAH activity (marked by treatment with DMSO; Fig. 2A) was found higher than MAGL activity with 2.3 ± 0.6 a.u. (*N* = 6) *vs.* 0.7 ± 0.3 a.u. (*N* = 6), respectively (*p* = 0.02, Mann Whitney U test; Fig. 2B). No sex difference was found in FAAH and MAGL profiles in human meninges (Fig. 2A and Additional file 2). FAAH activity was also assessed upon in vitro treatment with MAGL and FAAH inhibitors. This revealed that, comparably to what was observed in rat meninges, selective FAAH-inhibitor JZP-327A (1 µM) (in DMSO; taken as control) showed a visible reduction of FAAH activity compared to basal activity (Fig. 2A). Rather unexpectedly, potent MAGL-inhibitor, JJKK-048 (100 nM) decreased human FAAH activity (Fig. 2A) compared to basal activity. Of note, the more selective specific MAGL-inhibitor, KML-29 (1 µM) did not affect FAAH activity in human meninges. The dual MAGL/FAAH inhibitor AKU-005 (100 nM) significantly reduced FAAH basal activity to 61.6 ± 8.7% (*N* = 6,* p* = 0.002, Mann Whitney U test) in males and to 26.1 ± 6.2% (*N* = 6, *p* = 0.002, Mann Whitney U test) in females (Additional file 2) in comparison to FAAH basal activity (taken as 100%). Also, in human meninges the levels of 2-AG and AEA were measured and showed that the level of 2-AG was much higher than that of AEA (1.9 ± 0.98 pM/ng (*N* = 6) *vs.* 0.007 ± 0.003 pM/ng (*N* = 6), respectively (*p* = 0.002, Mann Whitney U test; Fig. 2C), similarly to the findings in rat meninges. Hence, despite some observed species differences, the results show that both rat and human MAGL/FAAH activity profiles, as well as rat and human 2-AG and AEA endoCB levels are comparable.

### Action of dual MAGL/FAAH inhibitor AKU-005 on meningeal afferents spiking in rats

**Fig. 3:**
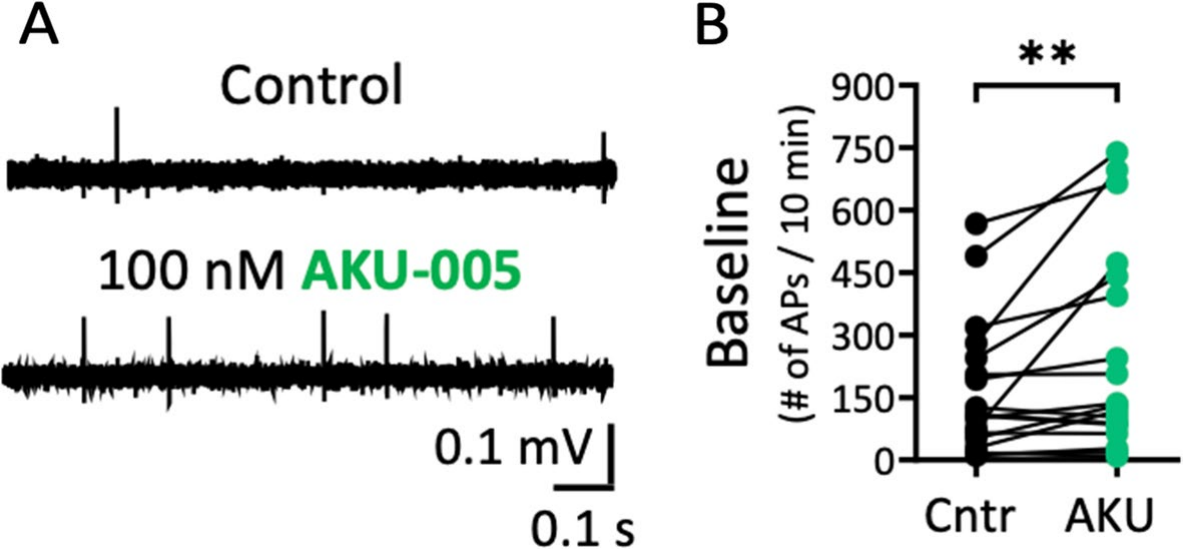
Effect of dual MAGL-FAAH inhibitor AKU-005 on rat meningeal fibres. **A** Example traces of control basal and AKU-005 (100 nM) induced activity in rat meningeal fibres. **B** Statistics show lightly increased general excitability in the presence of AKU-005 as evidenced by an increasing number of APs for the 10-min application window in the presence of AKU-005 (AKU) (*N* = 19, Wilcoxon test) compared to the control condition (Cntr) (*N* = 19, Wilcoxon test, ** = 0.002)

**Fig. 4:**
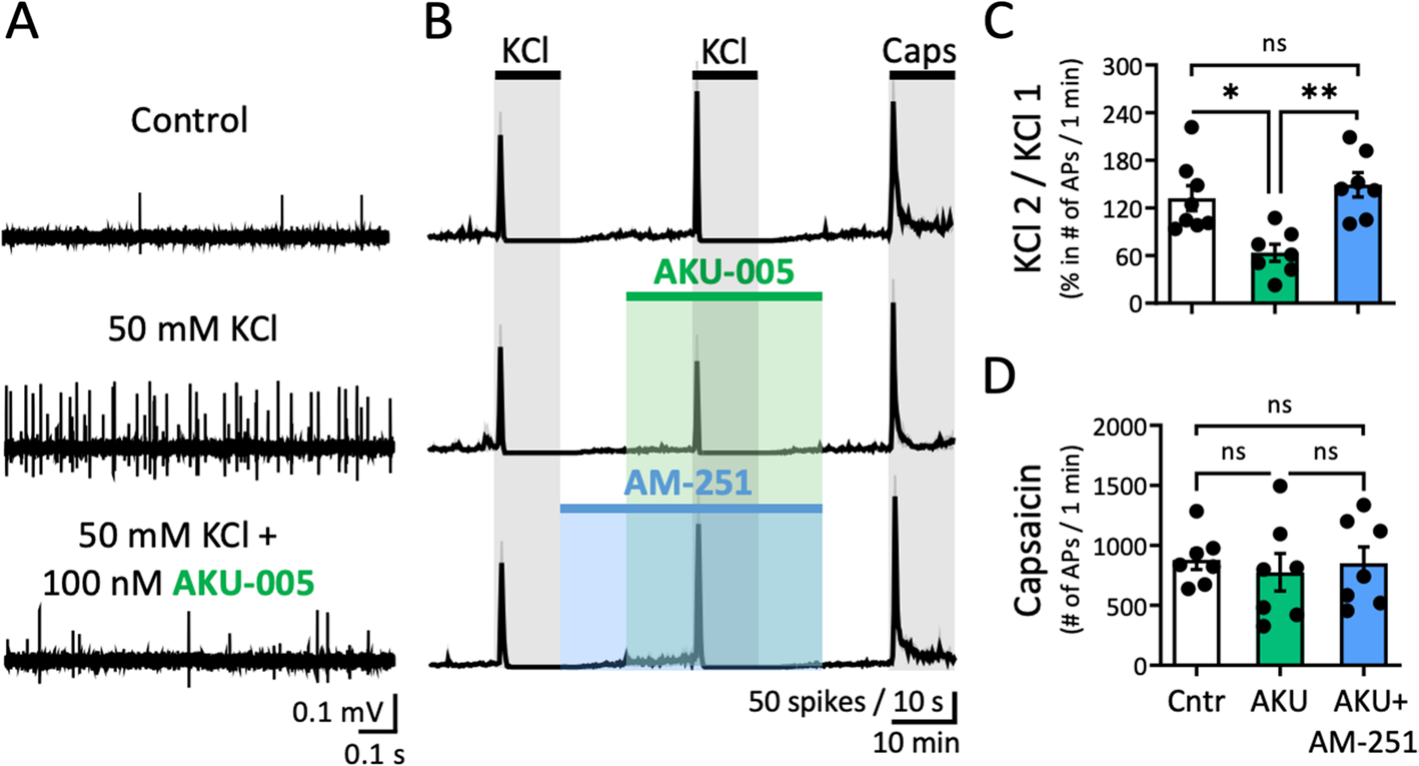
Dual MAGL/FAAH inhibitor AKU-005 reduces nociceptive firing mediated by CB1 receptors. **A** Example traces of APs recorded from the peripheral part of trigeminal nerve innervating rat meninges for the control condition before any application (top), during the 1-min active phase of KCl-induced spiking activity without (middle) and in the presence of AKU-005 (100 nM) (bottom trace). **B** Time courses of spike frequency (10-s bin size) induced by two subsequent 10-min pulses of 50 mM KCl and 1 μM Capsaicin, in the presence of only 100 nM AKU-005 and the combination of 100 nM AKU-005 and 1 μM AM-251 during the 2^nd^ KCl pulse. After exposure to AKU-005, the 2^nd^ KCl-induced firing was reduced. To test that the effect was mediated by cannabinoid CB1 receptors, the experiment was repeated in the presence of specific CB1 blocker AM-251 (1 µM). **C** Firing induced during the 2^nd^ KCl pulse was reduced in the presence of AKU-005 (AKU) application (*N* = 7, Kruskal Wallis test, * = 0.03) in comparison to the control (*N* = 8) condition (Cntr) (the ratio of the APs between 2^nd^ and 1^st^ KCL pulse). Firing was increased again by the combination of both AM-251 and AKU-005 (*N* = 7, Kruskal Wallis test, ** = 0.004). **D** Blockade of CB1 receptors did not change the efficacy of next applied capsaicin compared to application of only AKU-005 (number of APs/1 min)

Next, we tested the action of *dual* MAGL/FAAH inhibitor AKU-005 (AKU) on basal spiking activity of meningeal trigeminal afferents in rat hemiskulls. A slight but consistent increase in firing rate was detected after application of AKU-005 (100 nM) (Fig. 3A), which following experiments will explain to be linked to concomitant activation of CB1 and TRPV1 receptors by AEA enhancement. Within the 10-min window of application of this inhibitor, the number of APs increased from mean basal 199.6 ± 48.48 to 321.6 ± 85.25 (*N* = 19, *p* = 0.004, Wilcoxon test; Fig. 3B) after compound application. Subsequently, we tested if the increase of endoCB activity caused by dual MAGL/FAAH inhibition affected the excitability of meningeal nerve fibres upon application of 50 mM KCl twice (paired KCl pulses) added with a 20-min interval. The 2^nd^ depolarizing KCl pulse was administered after an intense washout of the 1^st^ pulse. At the end of the experiment, each preparation was tested with a fast capsaicin (1µM) application to activate TRPV1 receptors that could be also targeted by AEA. Figure 4A shows electrophysiological example traces taken from the 1-min active phase of the 2^nd^ KCl pulse (in DMSO) (control) in comparison to when also AKU-005 (100 nM) was applied. Paired pulses of only KCl revealed an enhancement of spiking activity for the 2^nd^ KCl pulse, indicating paired-pulse potentiation (PPP; Fig. 4B). The PPP was calculated as the ratio between the 2^nd^ and 1^st^ KCl (taken as 100%) pulse, here resulting in 132.2 ± 15.8% (*N* = 8) (control condition). When also AKU-005 (100 nM) was applied, this led to a reduced activation of meningeal nerves during the 2^nd^ KCl pulse, which can be interpreted as paired-pulse depression (PPD, calculated as the ratio between the 2^nd^ and 1^st^ KCl pulse, here resulting in 63.6 ± 10.8% (*N* = 7). This indicates a significant inhibitory effect of the dual inhibitor when compared to the control condition (*p* = 0.03, Kruskal–Wallis test; Fig. 4C). Next, to test the hypothesis that the reduced meningeal spiking activity effect was mediated by cannabinoid CB1 receptors, the main neuronal inhibitory receptor type typically mediating the analgesic effects of cannabinoids, we combined the application of AKU-005 with specific CB1 antagonist/inverse agonist AM-251 (1 µM) (Fig. 4B). Notably, an upregulation of spiking activity for the 2^nd^ KCl PPP response was seen when both AKU-005 and AM-251 were combined. Thus, the reduced response upon AKU-005 application was increased to 149.1 ± 15.3% (*N* = 7, *p* = 0.004, Kruskal–Wallis test; Fig. 4C) when also AM-251 was applied and reached the level of firing seen with the control condition. Finally, pro-nociceptive capsaicin-induced firing through TRPV1 channels was neither affected by prior application of AKU-005 or AKU-005 and AM-251 combined (Fig. 4D). Thus, inhibition of MAGL/FAAH activity by AKU-005 demonstrated a specific effect on the anti-nociceptive endoCBs enhancement that is mediated by CB1 receptor activity.

### Effect of exogenously applied 2-AG on spiking activity of meningeal afferents

**Fig. 5:**
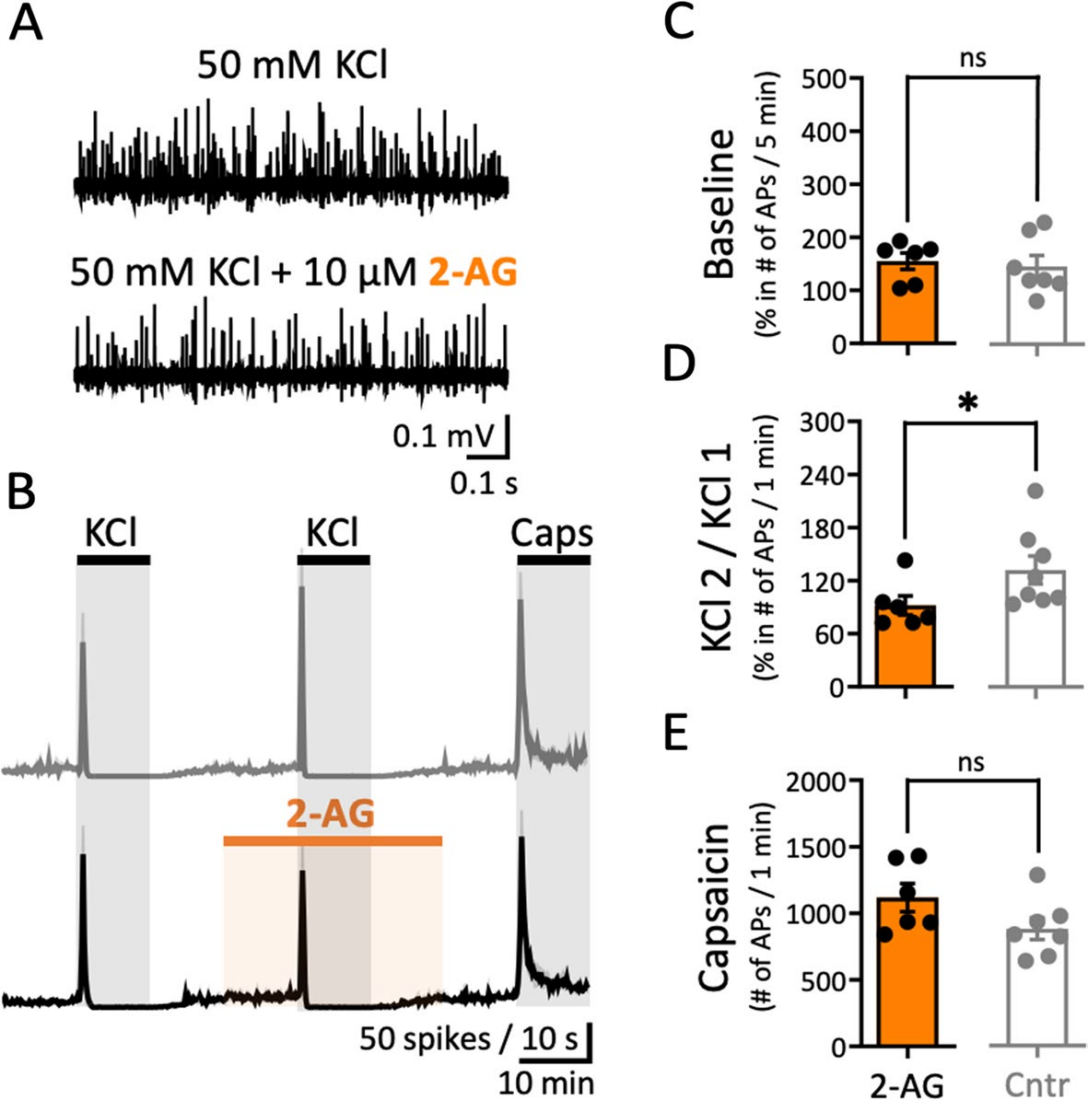
Exogenous 2-AG mildly reduces meningeal nerve endings nociceptive firing. **A** Example traces of APs recorded from the peripheral part of the rat trigeminal nerve within control, *i.e.* during the 1-min active phase of the 1^st^ KCl pulse and the corresponding phase of the 2^nd^ KCl pulse when also 10 µM 2-AG was present. Note the slight decrease of general nociceptive firing that was obtained during the combined application. **B** Time courses of spike frequency (10-s bin size) induced by paired pulses of KCl and 1 µM Capsaicin as control (data of the top panel in Fig. 4B) and during the combined application of 10 µM 2-AG with the 2^nd^ KCl pulse (bottom panel). **C** No difference was observed for the number of APs induced when comparing a ratio of the 5-min windows before/after application of 2-AG (*N*=6) to the same windows of the ratio spontaneous spiking activity in the control condition (Cntr, *N* = 7; data representation from Fig. 4B). **D** 2-AG application significantly reduced the number of APs during the 1-min active phase of the 2^nd^ KCl pulse compared to that of the 1^st^ KCl pulse. The ratio of APs (between 2^nd^ and 1^st^ (taken as 100%) KCl pulse) in the control (Cntr*, N* = 8; data representation from Fig. 4C) condition resulted higher than in the presence of 2-AG (*N* = 6, Mann Whitney test, * = 0.02). **E** No difference was observed for the number of APs during the 1-min active phase of 1 µM Capsaicin of the control condition (Cntr*, N* = 7; data representation from Fig. 4D) and after application of 2-AG (*N*=6)

Using the same control group with paired KCl pulses on rat meningeal afferents (Fig. 4), we also assessed the effects of exogenous 2-AG (10 µM) on excitability generated by the 2^nd^ KCl pulse (Fig. 5A and B). The action of pure exogenous 2-AG did not change the firing rate of basal spiking activity (Fig. 5C *vs*. control in Fig. 4). Instead of PPP observed for the whole nerve activity in control, a mild promotion to PPD was observed after 2-AG application to 92 ± 10.9% (*N* = 6, *p* = 0.02, Mann–Whitney U test; Fig. 5D *vs*. control Fig. 4C). The capsaicin-induced number of APs for the 1-min active phase after the 2-AG application was not affected (Fig. 5E *vs*. control Fig. 4D). To test the sex-dependency of endocannabinoids enhancement effect in meninges spiking activity, we similarly applied 2-AG (10 µM) on female rat meningeal afferents. Exogenously applied 2-AG did not change basal activity compared to control conditions in females as it did in males, but did show a mild inhibitory effect on nerve firing generated by the 2^nd^ KCl pulse, hence not significant (Additional file 3). Instead, action of capsaicin, operating via TRPV1 receptor signalling, remained unchanged, confirming that the 2-AG and TRPV1 pathways do not interact with each other.

### Effect of exogenously applied AEA on spiking activity of meningeal afferents

**Fig. 6:**
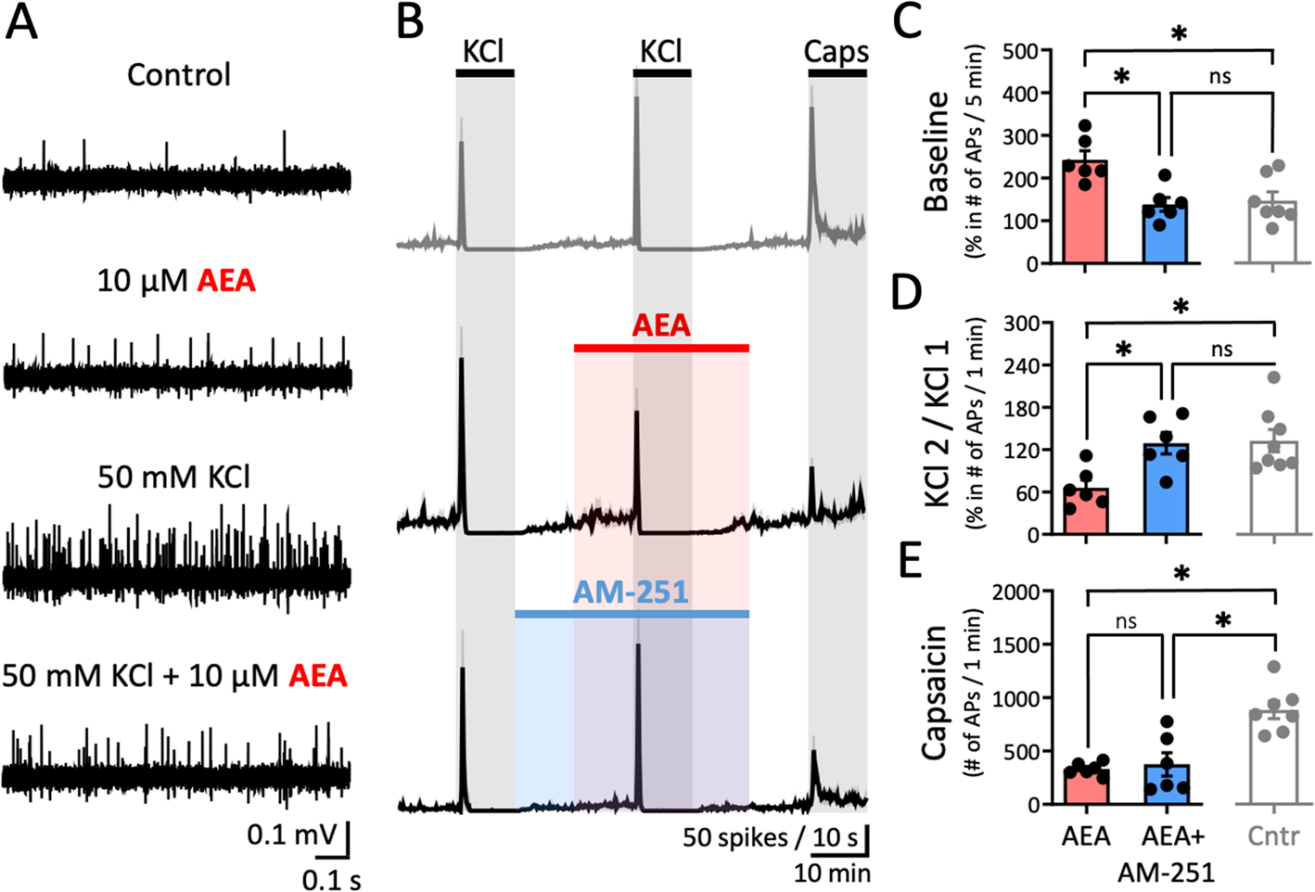
Strong reduction of general meningeal excitability by exogenous AEA. **A** Example traces of APs recorded from the peripheral part of trigeminal nerve innervating rat meninges for the control condition *i.e.* before any application, during application of 10 µM AEA, within the 1-min active phase of the 1^st^ KCl pulse and within the 1-min active phase of the 2^nd^ KCl pulse when also 10 µM AEA was present. Note the increased nociceptive firing during 10 µM AEA application that decreased during the combined application with KCl. **B** Time courses of spike frequency (10-s bin size) induced by two subsequent KCl pulses of 50 mM KCl and 1 µM Capsaicin in the control condition (data representation from Fig. 4B), within the combined application of 10 µM AEA with the 2^nd^ KCl pulse, and in the presence of CB1 antagonist AM-251 (1 µM). **C** The ratio before and during the first 5-min of the AEA application (*N* = 6) was lower compared to the corresponding windows ratio in the control condition (Cntr, *N* = 7, data representation from Fig. 4B; Kruskal Wallis test, * = 0.03) Combined application of AEA and AM-251 counteracted the effect (*N* = 6, Kruskal Wallis test, * = 0.02). **D** The ratio of KCl-evoked APs (between the 2^nd^ and the 1^st^ (taken as 100%) KCl pulse) within the 1-min active phase was strongly decreased from the control (Cntr, *N* = 8, data representation from Fig. 4C) condition when AEA was applied (*N* = 6, Kruskal Wallis test, * = 0.02). Application of also AM-251 counteracted the effect (*N* = 6, Kruskal Wallis test, * = 0.02). **E** The number of APs during the 1-min active phase of 1 µM Capsaicin was reduced during the AEA application (*N* = 6, Kruskal Wallis test, * = 0.01) and during the combined application of AEA and AM-251 (*N* = 6, Kruskal Wallis test, * = 0.01) compared to the control condition (Cntr*, N* = 7; data representation from Fig. 4D)

Next, we aimed to clarify whether exogenously applied AEA can affect peripheral meningeal nociception compared to the control group (Fig. 4). In addition to the strong inhibitory effect (PPD in the paired KCl pulse in control condition), a small excitatory effect presented as a transient increase of APs frequency during the first 5 min of application of 10 µM AEA (Fig. 6A). To investigate whether combined anti- and pro-nociceptive effects of AEA are mediated by CB1 receptors, we repeated the experiments in the presence of specific antagonist/inverse CB1 receptor agonist 1 µM AM-251 (Fig. 6B). For assessment of possible changes in AEA-evoked AP activity, we calculated the ratio of APs for an interval of 5 min before and during AEA, before and during a combination of AEA and AM-251, and compared results to the control group (data of the top panel in Fig. 4 B) with the same timeline ratio (Fig. 6C). The ratio was increased during the first 5 min of AEA application (242.8 ± 21%; *N* = 6) compared to that of the control group (data of the top panel in Fig. 4 B) (146.3 ± 20.8%, *N* = 7, *p* = 0.03, Kruskal–Wallis test; Fig. 6C). However, spiking activity recovered (138.3 ± 16.2%, *N* = 6, *p* = 0.02, Kruskal–Wallis test; Fig. 6C) when both in AEA + AM-251 were applied in comparison to only AEA. Figure 6B shows that application of AEA converted PPP into PPD comparing to the control condition (data of the top panel in Fig. 4B). PPP was however restored when AEA and AM-251 were both applied. Further analysis confirmed that AEA produced a strong PPD effect as evidenced by the reduced ratio between the paired KCl pulses (65.8 ± 11.1%, *N* = 6, *p* = 0.02, Kruskal–Wallis test; Fig. 6D) in comparison to PPP effect observed in control condition before (data from Fig. 4C). CB1 blockage by AM-251 restored PPP, *i.e.* the KCl ratio increased (129 ± 15.1% (*N* = 6, *p* = 0.02, Kruskal–Wallis test; Fig. 6D), which effectively eliminated the AEA anti-nociceptive effect. Notably, the number of capsaicin-induced APs in the 1-min active phase reduced from 880.6 ± 81.9 (*N* = 7) in the control condition (data from Fig. 4D) to 330.8 ± 24.5 following AEA application (*N* = 6, *p* = 0.01, Kruskal–Wallis test; Fig. 6E) and to 374.5 ± 109.6 (*N* = 6, *p* = 0.01, Kruskal–Wallis test; Fig. 6E) after application of both AEA and AM-251. As AEA also is an endogenous agonist of TRPV1 receptors, when administrated before capsaicin, it binds to TRPV1 and traces of such action of AEA could be a reason for the fluctuating neuronal activity visible after the sharp activity peak caused by capsaicin application (middle panel, Fig. 6B). Therefore, when capsaicin is later applied, TRPV1 receptors may already be either occupied or desensitized by AEA. Indeed, we observed that the response to TRPV1 receptors following AEA application is much smaller and unstable after the peak than the one traditionally provoked by capsaicin (data of the top panel in Fig. 4B). The specific role of AEA desensitizing TRPV1 receptors is distinct from 2-AG, which cannot interact with TRPV1 and, therefore, does not affect capsaicin response. Hence, AEA has a strong depressant effect on meningeal spiking even though it activates both anti-nociceptive CB1 and pro-nociceptive TRPV1 receptors. Notably, the observed effects of AEA on basal activity and KCl ratio as well as the effect of capsaicin after AEA on APs are similar in males and females (Additional file 3).

### Action of AEA via TRPV1 receptors

**Fig. 7:**
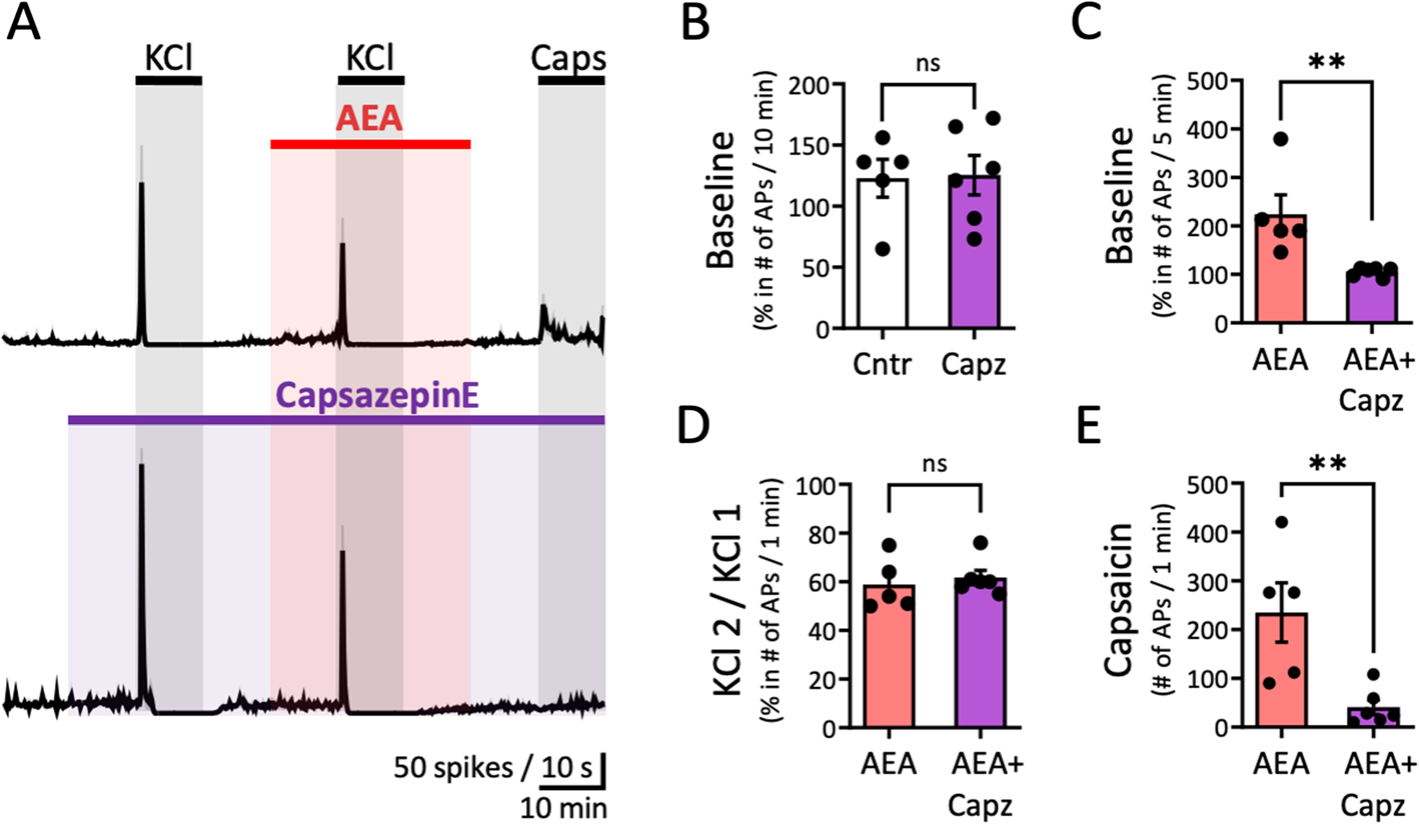
AEA analgesia and its interaction with TRPV1 receptors. **A** Time courses of spike frequency (10-s bin size) induced by APs recorded from the peripheral part of trigeminal nerve innervating rat meninges within the 1-min active phase of the 2^nd^ KCl pulse in combination with 10 µM AEA or with the co-application of 10 µM AEA and 20 µM capsazepine. Note the non-significant changes in nociceptive firing during applications of 10 µM AEA and the combination of 10 µM AEA and 20 µM capsazepine. Notably, capsaicin-induced firing decreased during the combined application of 10 µM AEA and 20 µM capsazepine. **B** No difference was observed between the APs ratio before and during 10 min capsazepine comparing to the Aps ratio of the same tine windows in the control condition. **C** The ratio of APs (before/during application of exogenous AEA, *N* = 5) for the 5-min baseline returned to the control baseline level in presence of capsazepine (*N* = 6, Mann Whitney U test, ** = 0.004). **D** The percentage between the number of APs induced by the 1^st^ and 2^nd^ KCl pulse for the 1-min within AEA was not affected by the application of both 10 µM AEA and 20 µM capsazepine. **E** The number of APs during the 1-min active phase of 1 µM Capsaicin when also AEA was applied (*N*=5) was reduced after application of both 10 µM AEA and 20 µM capsazepine (*N* = 6, Mann Whitney U test, ** = 0.008)

To further explore the dual agonism of AEA on CB1 and TRPV1 receptors, we next investigated the effect of AEA (10 µM) on the ratio of the paired KCl pulses when also capsazepine (20 µM), a specific TRPV1 antagonist, was applied (Fig. 7A). Capsazepine was applied throughout the recording period to detect capsazepine-dependent changes. Analysis of the data indicated that capsazepine itself did not alter spontaneous APs as the ratio of APs (before/during capsazepine only application) for the 10-min period was not affected when compared to the same time period in the control condition (Fig. 7B). However, capsazepine treatment reduced the AEA-evoked response through TRPV1 receptors, which was observed before peak firing. The ratio of APs (before/during application of only exogenous AEA) for a 5-min baseline (223.6 ± 40.3%, *N* = 5) *vs*. the same time period (before/during AEA + capsazepine) was restored to baseline (105.5 ± 3.8%, *N* = 6, *p* = 0.004, Mann Whitney U test; Fig. 7C). Thus, the increase of meningeal spiking by AEA within the first 5 min of application mostly activates TRPV1 receptors. Instead, the following AEA depressant effect due to CB1 activation remained unchanged, *i.e.* it reduced KCl-induced excitability. In line with these results, the ratio of the paired KCl evoked APs was similar for the application of AEA alone and the combined application of AEA and capsazepine (Fig. 7D). Focusing on the capsaicin-induced TRPV1 response, it is clear that capsazepine strongly reduced the number of capsaicin-evoked APs within the 1-min active phase (234.8 ± 60.7 (*N* = 5) *vs.* 40.7 ± 15.1 (*N* = 6)), proving that it can effectively block TRPV1 channels (*p* = 0.008, Mann Whitney U test; Fig. 7E). These experiments show that capsazepine is capable of preventing both capsaicin- and AEA-evoked TRPV1 responses.

### Cluster analysis of endoCBs signalling in the meninges

**Fig. 8:**
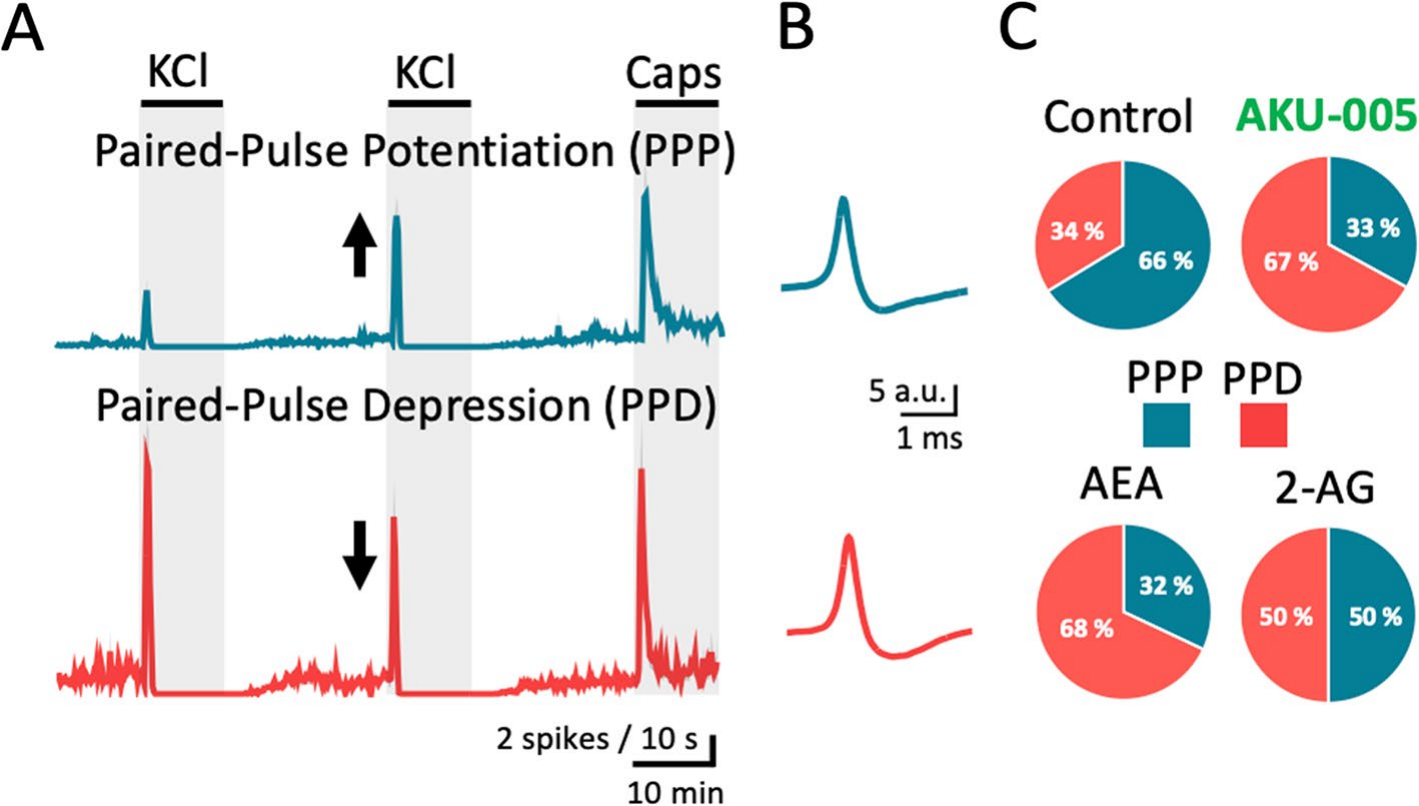
AKU-005 and AEA promote paired-pulse depression in meningeal afferents. **A** Time courses of spike frequency (10-s bin size) induced by APs recorded from the peripheral part of trigeminal nerve innervating rat meninges within the 1-min active phase of the 2^nd^ KCl pulse. By clustering the recordings, we defined paired-pulse potentiation (PPP) when the number KCl-evoked APs was higher during the 2^nd^ KCl pulse and paired-pulse depression (PPD) when the number KCl-evoked APs was higher during the 1^st^ KCl pulse. **B** AP morphologies corresponding to PPP and PPD recordings showed no differences. **C** A statistical analysis showed that physiologically PPP are more frequent than PPD. In the presence of AKU-005 or AEA during the 2^nd^ KCl pulse, PPD clusters are more prevalent for AKU-005 and AEA, while PPP clusters are less prevalent under the same treatments. PPP and PPD clusters are comparable when 2-AG was applied during the 2^nd^ KCl pulse

Cluster analysis allowed the exploration of the action of endoCBs at the level of single fibres (or small groups of similar fibres) [30]. When analysing the firing from trigeminal meningeal afferents, we identified two opposite type of responses of fibres contributing either to the facilitatory effect presented as PPP or the inhibitory PPD effect (Fig. 8A). In the control condition, 66% of the fibres revealed PPP, whereas PPD was observed for 34% of the fibres (Fig. 8C). However, in experiments with application of either AKU-005 or AEA, the majority of the fibres revealed PPD, *i.e.* 67% for AKU-005 and 68% for AEA (Fig. 8C). Fibres revealing PPP where only 33% for AKU-005 and 32% for AEA (Fig. 8C). In contrast, when applying 2-AG fibres revealing PPP and PPD were equally prevalent (50%/50%; Fig. 8C), suggesting a higher efficacy of AEA as a local inhibitor. Notably, the shape of the APs was similar for both type of responses of these fibres (Fig. 8B), suggesting that both phenomena developed in the similar types of meningeal afferents. Together, the analysis was consistent with the more prominent role of the enhancement of the anti-nociceptive AEA by AKU-005 treatment in the action of dual MAGL/FAAH inhibition in meninges.

## Discussion

Here we show, for the first time, that in rat and human meninges basal FAAH activity is high compared to hardly detectable MAGL activity. Such pronounced FAAH activity in meninges is in a sharp contrast to our earlier observed high MAGL/low FAAH activity in trigeminal ganglia and dorsal root ganglia [20]. Notably, both meningeal nerve fibres and the neuroglial system in trigeminal ganglia contribute to migraine pain [1, 2], but as we here show seem to have opposing activity profiles. In addition, we show that both the anti-nociceptive main endoCBs 2-AG and AEA significantly reduced KCl-evoked spiking in meningeal afferents, suggesting that engaging multi-endoCB anti-nociceptive action in both the meninges and the trigeminal ganglia may provide a more effective inhibition of headache mechanisms in migraine pathology.

### AEA and 2-AG have complementary roles in meningeal anti-nociception

It was earlier proposed that MAGL inhibition elevated 2-AG signalling in the trigeminovascular system and thereby plays a role in decreasing nociception in migraine [20]. Our current study is in line with this and shows that under basal conditions, the concentration of 2-AG in the meninges is much higher than that of AEA, likely due to a higher activity of FAAH compared to that of MAGL in this peripheral area. However, we also found that the application of exogenous 2-AG produced only a mild inhibitory effect in meningeal afferents upon excitation with KCl, suggesting that signalling by this MAGL substrate is only moderate. AEA also is a CB1 receptor agonist, albeit with lower potency and efficacy [35]. Notably, although in control conditions the basal level of AEA is much lower than that of endogenous 2-AG, inhibition of meningeal APs by exogenous AEA administration was more pronounced than that of applied 2-AG. Given the high basal activity of FAAH in the meninges, the amount of endogenous AEA could be largely increased after inhibition of FAAH, the AEA degrading enzyme, and could therefore have therapeutic potential. Moreover, we found that KCl treatment doubles AEA levels in rat meninges per se. The KCl-induced depolarization could determine a traditional release on demand of AEA from the presynaptic terminals of neurons to compensate for the induced meningeal spiking activity. Consistent with this, a recent finding suggests that elevated AEA signalling by FAAH inhibition in meninges was shown to have a significant anti-nociceptive effect [36]. The primary cell types that are capable of synthesizing endocannabinoids are nerve cells, glial and immune cells [17, 37]. Thus, AEA could be released in the meninges in our assays, from these cellular sources. In addition to abundant neurons in the meninges, local immune cells are responding to transmitters released from depolarized local neurons could largely participate in secretion of endocannabinoids. Notably, aside of activating CB1 receptors, both 2-AG and AEA can also activate CB2 receptors expressed on immune cells [38]. The degranulation of mast cells in meninges, which express CB2 receptors [38–40], may therefore contribute to the sensitization and activation of meningeal afferents [41].Nonetheless, we show that meningeal CB1 receptors play an important role in exerting the depressant effect of endoCBs on meningeal spiking, as it can be fully reversed by application of CB1 antagonist AM-251.

### Dual MAGL/FAAH inhibitor AKU-005 is promising in reducing migraine nociception

FAAH appears to be the only active endoCB hydrolase in the meninges. Therefore, it seems a promising strategy to specifically target FAAH in that tissue. Indeed, specific FAAH-inhibitors URB-597 and URB-937 were shown to reduce nitroglycerin (NTG)-induced trigeminal hyperalgesia [42, 43]. Furthermore, the very good selectivity of FAAH inhibitors makes them very attractive for pain treatment [39, 44]. Whereas there are ongoing efforts to specifically inhibit either FAAH or MAGL, we here promote the use of a *dual* inhibitor that targets both endoCB degrading enzymes [45]. More specifically, given the opposite activity profiles and dominant roles FAAH in meninges and MAGL in trigeminal ganglia, both areas involved in migraine nociception [20], we show that the combined inhibition of MAGL and FAAH enzymes to degrade 2-AG and AEA, respectively, has a greatly added benefit [20]. In support of our line of reasoning, dual inhibitor JZL-195 was also recently shown effective in relieving inflammatory meningeal pain and reducing trigeminal hyperalgesia [46–49]. However, neither JZL195 nor AKU-005 were tested in the context of migraine pathology in meningeal tissues, especially in human meninges. Indeed, it was shown that JZL195 in human brain tissue could be distinctly sensitive to MAGL/FAAH inhibitors compared to activity in rats (up to 20 times) [50]. Instead, we show in this study, that FAAH inhibition by AKU-005 is similarly behaving and with even a higher potency in human dura compared to that in rat meninges. Moreover, previous papers on JZL195 in mouse models of neuropathic pain and migraine-like nitroglycerin rat models [46–49] did not provide a mechanistical explanation behind the observed anti-nociception. Here we show a functional beneficial effect on KCl-induced spiking activity in meninges of the developed potent dual MAGL/FAAH inhibitor AKU-005 [19].

One interesting point which remains unclear if endocannabinoid receptors are located purely at the nerve endings of meningeal afferents or expressed along the whole nerve fibres. Consistent with the latter, others and our group showed calcium-dependent release events along the nerve axons [51, 52] and location of ATP-, serotonin- as well capsaicin-gated receptors in the meningeal nerve fibres disconnected from periphery [53]. Similarly, it has been proposed that CGRP receptors are located in the nodes of Ranvier [54]. In this study, we showed that, under basal conditions, AKU-005 produced only minor firing of meningeal fibers In contrast, when meningeal firing is increased, by for instance application of KCl as done for the present study, thereby mimicking the increased firing relevant to migraine, AKU-005 drastically reduced meningeal firing. Moreover, using CB1-specific antagonist AM-251, we could demonstrate that AKU-005’s inhibitory effect was mediated by CB1 receptors that are known to have an anti-nociceptive role [55, 56]. AKU-005 potency is relevant for both MAGL and FAAH inhibition in rat and human brain membranes [19], which confirms the translational power of this compound. Further studies on the docking properties of AKU-005 to human specific areas will reveal a better profiling of the selective effects of this compound in different migraine nociceptive processes. However, in the context of a distinct profile of MAGL and FAAH activity in areas involved in migraine pain signalling [20], MAGL prevails over FAAH in most of the areas with the exception of the meninges. Therefore, the dual MAGL/FAAH inhibitor AKU-005, which inhibits both serine hydrolases, although being more potent for MAGL over FAAH [19], represents the perfect fit to target the whole trigeminal nociceptive system including meninges and trigeminal ganglia.

### AEA: an endogenous compound with pro- or anti-nociceptive properties?

Our cluster analysis revealed that the paired KCl protocol generated short-lasting bursts of spiking activity in the meninges that were more pronounced with the 2^nd^ KCl pulse, hence referred to as “paired-pulse potentiation (PPP)”. Notably, a relative loss of excitability for the 2^nd^ KCl pulse compared to the 1^st^, referred to as “paired-pulse depression (PPD)”, was observed when AEA action in the meninges was enhanced, either by exogenous application of AEA or by raising its level by application of AKU-005. Along with the major depressant effect of AEA on spiking of meningeal afferents, our electrophysiological recordings resulted in a transient excitatory response of this endoCB on TRPV1 receptors. Of note, aside from acting on CB1 receptors, AEA also acts as an agonist of TRPV1 receptors expressed on trigeminal neurons [57, 58]. This effect can not only explain the transient moderate level of spiking induced by AEA, but also the minor tonic increase in basal firing of meningeal nerve fibres provoked by AKU-005. Notably, the raised level of AEA (but not 2-AG) was able to reduce the response to a subsequent application of the pro-nociceptive capsaicin. Whether this reduction in capsaicin response is due to occupied binding sites or desensitization remains an open question. It has been, however, suggested that most likely AEA activates TRPV1 receptors at a different location that the one where capsaicin binds [59]. Therefore, the latter response was most likely dependent on desensitization of TRPV1 receptors, which are activated by AEA but not 2-AG. Thus, AEA reduced the meningeal spiking activity induced by two independent pro-nociceptive stimuli: KCl and capsaicin. Our data suggest that the reducing effect of AEA on KCl-induced APs was mainly mediated by CB1 receptors, as it was insensitive to TRPV1 antagonist capsazepine. Nevertheless, the dualism of interactions of AEA with TRPV1 receptors should be taken into consideration when considering treatment options based on the inhibition of FAAH.

## Conclusion

Taken together, our findings suggest that the AEA/FAAH pathway holds a promise for inhibiting migraine-related pathological meningeal neuronal hyperexcitability via CB1 receptors and potentially affecting TRPV1 receptors. Whereas the high basal concentration of endoCB 2-AG in meninges may complement local anti-nociceptive effect, AEA appears to be even a more powerful and tuneable peripheral inhibitor of meningeal afferent firing. In conclusion, dual MAGL/FAAH inhibitor AKU-005 represents a promising compound to counteract migraine nociception, which originates from the trigeminal nociceptive system that includes the meninges.

## Supplementary Information

**Additional file 1. **Competitive gel-basedABPP shows partial FAAH inhibition in rat female meninges by AKU-005. 

Additional file 2. Competitive gel-based ABPP shows dual MAGL/FAAHinhibitor AKU-005 potent in human meninges.

Additional file 3. Endocannabinoids 2-AG and AEA modulateKCl-induced spiking from meningeal afferents in female rats.

## Data Availability

The data sets generated and analysed for the study are available from the corresponding author on reasonable request.

## Abbreviations

2-AG: 2-Arachidonoylglycerol
ABPP: Activity-based protein profiling
aCSF: Artificial cerebrospinal fluid
AEA: Anandamide
APs: Action potentials
a.u.: Arbitrary units
BCA: Bicinchonic acid
CB1/2: Cannabinoid receptor type 1/2
DMSO: Dimethylsulfoxyde
endoCBs: Endocannabinoids
FAAH: Fatty acid amid hydrolase
KCl: Potassium chloride
LC–MS/MS: Chromatography coupled with triple quadrupole mass spectrometry
m: Mean
MAGL: Monoacylglycerol lipase
PBS: Phosphate-buffered saline
PPD: Paired-pulse depression
PPP: Paired-pulse potentiation
RT: Room temperature
SD: Standard deviation
SEM: Standard error of the mean
TAMRA-FP: Carboxytetramethylrhodamine-ActivX™ Fluorophosphonate
TRPV1: Transient receptor potential vanilloid 1
