# Supplementary material for: Potent dual MAGL/FAAH inhibitor AKU-005 engages endocannabinoids to diminish meningeal nociception implicated in migraine pain
Source: J Headache Pain. 2023 Apr 11;24(1):38. doi: 10.1186/s10194-023-01568-3 (PMC10088116; doi:10.1186/s10194-023-01568-3)
Supplement: Supplementary file 1 — Additional file 1. Competitive gel-basedABPP shows partial FAAH inhibition in rat female meninges by AKU-005. [file 10194_2023_1568_MOESM1_ESM.docx]

**
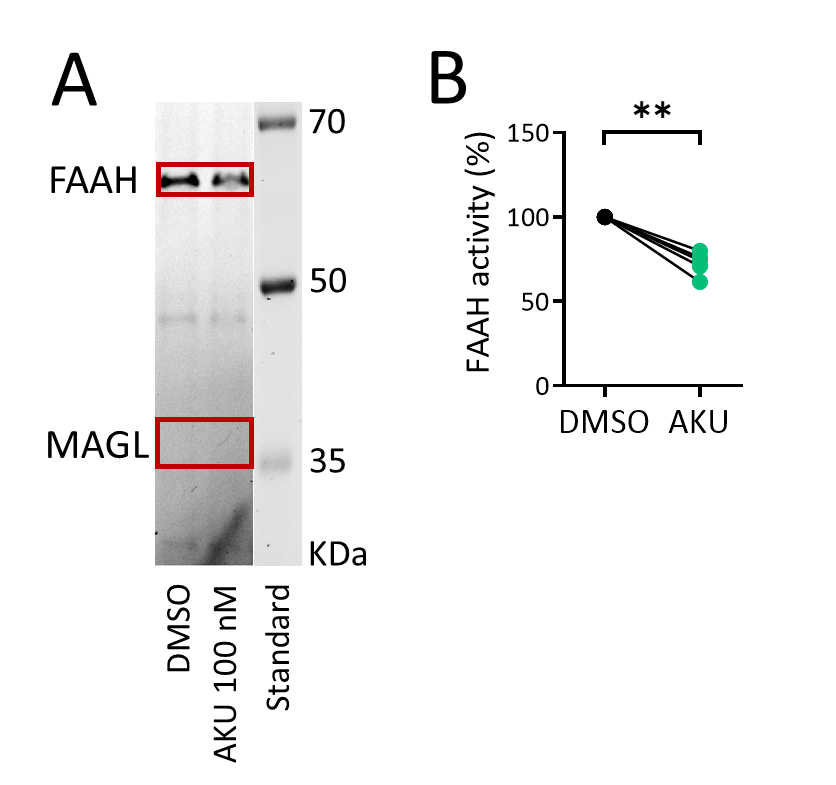
Supplementary Information**

**Additional File 1. Competitive gel-based ABPP shows partial FAAH inhibition in rat female meninges by AKU-005.** (**A**) Rat meninges were pre-incubated for 1 h with DMSO (vehicle) or with dual MAGL/FAAH-inhibitor - AKU-005 (AKU, 100 nM), and then labelled with fluorescent probe TAMRA-FP, as indicated in the Material and Methods. TAMRA-FP-labelled bands appear dark after in-gel imaging. FAAH and MAGL were identified based on selective inhibition and their expected molecular weights. MAGL and FAAH band-intensities after DMSO treatment represent basal MAGL and FAAH activities, respectively. Note that FAAH activity after DMSO treatment is high, whereas basal MAGL activity is practically absent. Basal FAAH- activity was visibly partially reduced with AKU-005. (**B**) Statistics comparing the FAAH basal activity with FAAH activity after 100 nM AKU-005 inhibition in rat meninges. FAAH basal activity (taken as 100%) was reduced by AKU-005 (AKU) inhibition (*N* = 6, Mann Whitney U test, ** = 0.002).
