# Supplementary material for: Potent dual MAGL/FAAH inhibitor AKU-005 engages endocannabinoids to diminish meningeal nociception implicated in migraine pain
Source: J Headache Pain. 2023 Apr 11;24(1):38. doi: 10.1186/s10194-023-01568-3 (PMC10088116; doi:10.1186/s10194-023-01568-3)
Supplement: Supplementary file 2 — Competitive gel-based ABPP shows dual MAGL/FAAHinhibitor AKU-005 potent in human meninges. [file 10194_2023_1568_MOESM2_ESM.docx]

**
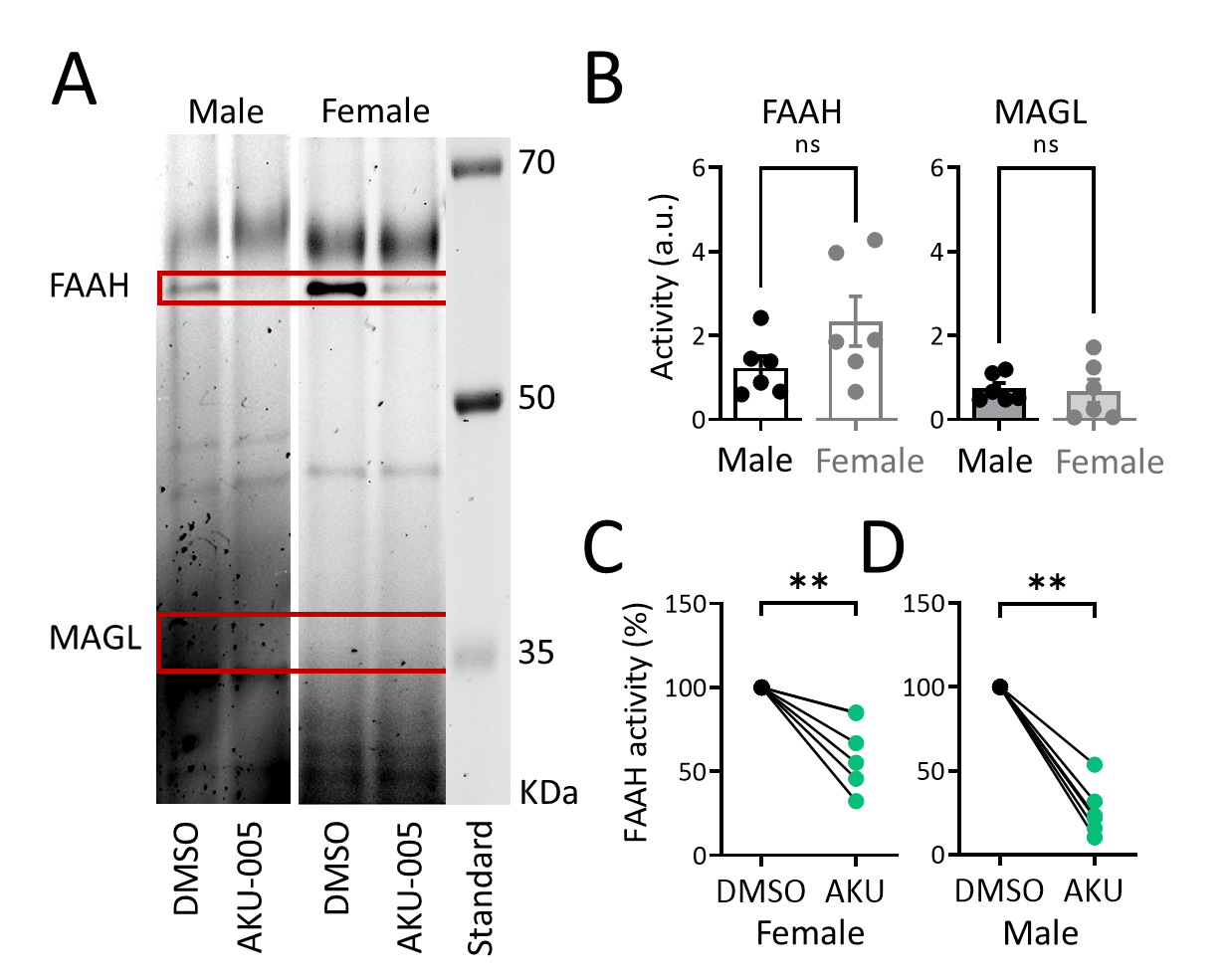
Additional File 2. Competitive gel-based ABPP shows dual MAGL/FAAH inhibitor AKU-005 potent in human meninges.** (**A**) Human meninges were pre-incubated for 1 h with DMSO (vehicle) and dual MAGL/FAAH inhibitors AKU-005 (100 nM), and then labelled with fluorescent probe TAMRA-FP, as indicated in the Material and Methods. TAMRA-FP-labelled bands appear dark after in-gel imaging. FAAH and MAGL were identified based on selective inhibition and their expected molecular weights. MAGL and FAAH band-intensities after DMSO treatment represent basal MAGL and FAAH activities, respectively. Note that FAAH activity after DMSO treatment is high whereas basal MAGL activity is practically absent. Basal FAAH activity in females appears higher than in males. Basal FAAH- activity reduces with AKU-005 pretreatment. (**B**) Statistics comparing the basal activity (DMSO) of MAGL and FAAH in male to female human meninges previously shown in Fig. 2 B. No sex difference was found in FAAH and MAGL profiles (N = 6 per sex, Kruskal Wallis test). (**C**) Statistics comparing the FAAH basal activity (DMSO) with FAAH activity after AKU-005 100 nM inhibition in female human meninges. FAAH basal activity (taken as 100%) was reduced by AKU-005 inhibition (N = 6 per sex, Mann Whitney U test, ** = 0.002). (**D**) Statistics comparing the FAAH basal activity with FAAH activity after AKU-005 100 nM inhibition in male human meninges. FAAH basal activity (taken as 100 %) was reduced by AKU-005 inhibition (N = 6 per sex, Mann Whitney U test, ** = 0.002).
