# Supplementary material for: Potent dual MAGL/FAAH inhibitor AKU-005 engages endocannabinoids to diminish meningeal nociception implicated in migraine pain
Source: J Headache Pain. 2023 Apr 11;24(1):38. doi: 10.1186/s10194-023-01568-3 (PMC10088116; doi:10.1186/s10194-023-01568-3)
Supplement: Supplementary file 3 — Endocannabinoids 2-AG and AEA modulateKCl-induced spiking from meningeal afferents in female rats. [file 10194_2023_1568_MOESM3_ESM.docx]

**
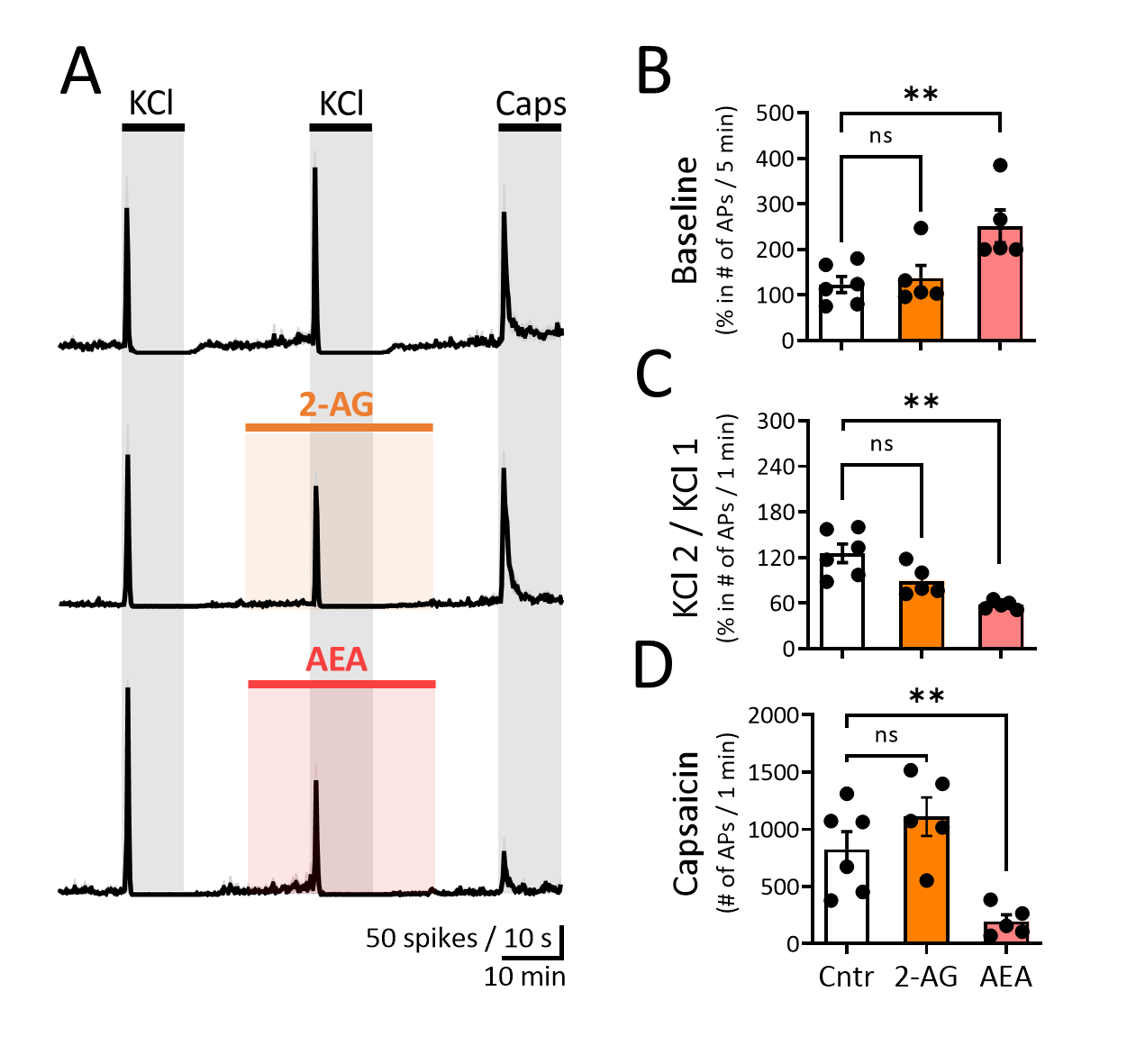
**

**Additional File 3. Endocannabinoids 2-AG and AEA modulate KCl-induced spiking from meningeal afferents in female rats.**

**(A)** Time courses of spike frequency (10-s bin size) induced by APs recorded from the peripheral part of trigeminal nerve innervating female rat meninges within the 1-min active phase of the 2^nd^ KCl pulse in combination with 10 µM AEA or 10 µM 2-AG. Note changes in KCL-induced firing during applications of 2-AG and AEA. Notably, capsaicin-induced firing decreased during the combined application of 10 µM AEA, but not during 2-AG. (**B**) No difference was observed between the APs ratio before and during 5 min 2-AG (N = 5), although it was between the APs ratio before and during 5 min AEA (N = 5) comparing to the APs ratio of the same tine windows in the control condition (Cntr, N = 6, Mann Whitney U test, ** = 0.004). (**C**) The ratio of APs for 1 min between the 2^nd^ and 1^st^ KCl pulses was affected by both endocannabinoids in comparison to control conditions (N = 6), but significantly only with 10 µM AEA (N = 5, Mann Whitney U test, ** = 0.004). (**D**) The number of APs during the 1-min active phase of 1 µM Capsaicin was reduced after application of 10 µM AEA only (N = 5, Mann Whitney U test, ** = 0.008).
